# A Functional Polymorphism Downstream of Vitamin A Regulator Gene *CYP26B1* Is Associated with Hand Osteoarthritis

**DOI:** 10.3390/ijms24033021

**Published:** 2023-02-03

**Authors:** Vivia Khosasih, Kai-Ming Liu, Chung-Ming Huang, Lieh-Bang Liou, Ming-Shium Hsieh, Chian-Her Lee, Chang-Youh Tsai, San-Yuan Kuo, Su-Yang Hwa, Chia-Li Yu, Chih-Hao Chang, Cheng-Jyh Lin, Song-Chou Hsieh, Chun-Ying Cheng, Wei-Ming Chen, Liang-Kuang Chen, Hui-Ping Chuang, Ying-Ting Chen, Pei-Chun Tsai, Liang-Suei Lu, Weng-Siong H’ng, Yanfei Zhang, Hsiang-Cheng Chen, Chien-Hsiun Chen, Ming Ta Michael Lee, Jer-Yuarn Wu

**Affiliations:** 1 Taiwan International Graduate Program in Molecular Medicine, National Yang Ming Chiao Tung University and Academia Sinica, Taipei 115, Taiwan; 2 National Center for Genome Medicine, Institute of Biomedical Sciences, Academia Sinica, Taipei 115, Taiwan; 3 Division of Immunology and Rheumatology, Department of Internal Medicine, China Medical University Hospital, Taichung 404, Taiwan; 4 Graduate Institute of Integrated Medicine, College of Chinese Medicine, China Medical University, Taichung 404, Taiwan; 5 Division of Rheumatology, Allergy and Immunology, New Taipei Municipal Tucheng Hospital, New Taipei City 236, Taiwan; 6 College of Medicine, Chang Gung University, Taoyuan 333, Taiwan; 7 Department of Orthopedics, School of Medicine, College of Medicine, Taipei Medical University, Taipei 110, Taiwan; 8 Department of Orthopedics, Taipei Medical University Hospital, Taipei 110, Taiwan; 9 Department of Orthopedics, En Chu Kong Hospital, New Taipei 237, Taiwan; 10 School of Medicine, National Yang Ming Chiao Tung University, Taipei 112, Taiwan; 11 Division of Allergy, Immunology and Rheumatology, Department of Medicine, Taipei Veterans General Hospital, Taipei 112, Taiwan; 12 Division of Rheumatology, Immunology and Allergy, Department of Internal Medicine, Tri-Service General Hospital, National Defense Medical Center, Taipei 114, Taiwan; 13 Department of Orthopaedics, Tri-Service General Hospital, National Defense Medical Center, Taipei 114, Taiwan; 14 Department of Internal Medicine, National Taiwan University Hospital, Taipei 100, Taiwan; 15 Institute of Molecular Medicine, College of Medicine, National Taiwan University, Taipei 100, Taiwan; 16 Department of Orthopedics, College of Medicine, National Taiwan University and National Taiwan University Hospital, Taipei 100, Taiwan; 17 Department of Orthopedics, National Taiwan University Hospital Jin-Shan Branch, New Taipei City 208, Taiwan; 18 Department of Orthopedics, China Medical University Hospital, Taichung 404, Taiwan; 19 Division of Allergy, Immunology, and Rheumatology, Department of Internal Medicine, National Taiwan University Hospital, Taipei 100, Taiwan; 20 Department of Orthopedic, Chang Gung Memorial Hospital, Linkou, Taoyuan 333, Taiwan; 21 Department of Orthopaedics and Traumatology, Taipei Veteran General Hospital, Taipei 112, Taiwan; 22 Department of Diagnostic Radiology, Shin Kong Wu Ho-Su Memorial Hospital, Taipei 111, Taiwan; 23 Genomic Medicine Institute, Geisinger, Danville, PA 17822, USA; 24 School of Chinese Medicine, China Medical University, Taichung 404, Taiwan

**Keywords:** hand osteoarthritis, *CYP26B1*, retinoic acid, Han Chinese, genome-wide association study, polymorphisms

## Abstract

While genetic analyses have revealed ~100 risk loci associated with osteoarthritis (OA), only eight have been linked to hand OA. Besides, these studies were performed in predominantly European and Caucasian ancestries. Here, we conducted a genome-wide association study in the Han Chinese population to identify genetic variations associated with the disease. We recruited a total of 1136 individuals (n = 420 hand OA-affected; n = 716 unaffected control subjects) of Han Chinese ancestry. We carried out genotyping using Axiom Asia Precisi on Medicine Research Array, and we employed the RegulomeDB database and RoadMap DNase I Hypersensitivity Sites annotations to further narrow down our potential candidate variants. Genetic variants identified were tested in the Geisinger’s hand OA cohort selected from the Geisinger MyCode community health initiative (MyCode^®^). We also performed a luciferase reporter assay to confirm the potential impact of top candidate single-nucleotide polymorphisms (SNPs) on hand OA. We identified six associated SNPs (*p*-value = 6.76 × 10^−7^–7.31 × 10^−6^) clustered at 2p13.2 downstream of the *CYP26B1* gene. The strongest association signal identified was rs883313 (*p*-value = 6.76 × 10^−7^, odds ratio (OR) = 1.76), followed by rs12713768 (*p*-value = 1.36 × 10^−6^, OR = 1.74), near or within the enhancer region closest to the *CYP26B1* gene. Our findings showed that the major risk-conferring CC haplotype of SNPs rs12713768 and rs10208040 [strong linkage disequilibrium (LD); D’ = 1, r^2^ = 0.651] drives 18.9% of enhancer expression activity. Our findings highlight that the SNP rs12713768 is associated with susceptibility to and severity of hand OA in the Han Chinese population and that the suggested retinoic acid signaling pathway may play an important role in its pathogenesis.

## 1. Introduction

Hand osteoarthritis (OA) is the most prevalent joint disease characterized by cartilage degeneration, bone sclerosis, and osteophytes, which manifests with loss of hand motion [1,2]. The disease affects different hand joints in a bilateral manner, frequently affecting the distal interphalangeal (DIP) and proximal interphalangeal (PIP) joints, the Heberden’s and Bouchard’s nodes, respectively, and the first carpometacarpal (CMC-1) joint [2,3]. The prevalence of hand OA differs by ethnicity [4,5,6,7,8]; however, most genetics studies of hand OA were conducted in European cohorts. In radiographic hand OA studies of Japanese [9] and Korean [10] populations, a higher prevalence of OA in the interphalangeal (IP) and a lower prevalence in the CMC thumb joints were reported when compared to those in Caucasian populations. Furthermore, previous studies have indicated that OA susceptibility loci identified in the European population are often not in accordance with Asian populations [11,12,13]. Hand OA is a multifactorial disease where age, sex, occupational activities, and genetics strongly contribute to the development and progression of the disease, which complicates efforts to identify pathogenic mechanisms [1,3].

Family-based and twin pair studies strongly suggest the genetic component of hand OA, with heritability estimates of ~64% [14,15]. Early genome-wide linkage and association studies have identified multiple hand OA susceptibility genes, such as *AGC1* [16,17,18], *HFE* [19,20], *GDF5* [12], and *A2BP1* [21]; however, neither of those studies show conclusive results, nor do they reach the genome-wide significance *p*-value. The first genome-wide association studies (GWASs) of hand OA to report genome-wide significant results identified a single-nucleotide polymorphism (SNP) rs3204689 at chromosome 15q22 (*p*-value = 3.99 × 10^−10^, odds ratio (OR) = 1.51) [22]. This SNP is mapped to the *ALDH1A2* gene that encodes retinaldehyde dehydrogenase 2 enzyme, which is involved in the conversion of retinoic acid during vitamin A metabolism [22]. Two other hand-OA associated genes have since been reported, *MGP* (*p*-value = 1.8 × 10^−15^) [23] and *WNT9A* (*p*-value = 2.4 × 10^−13^) [24]. The most recent large-scale meta-analyses of 177,517 individuals with OA (21,186 cases of hand OA) further identified 52 novel loci across 11 OA phenotypes, seven of which are associated with hand OA [25]. Most of these seven identified loci are annotated to the intron region, except for rs8112559, which is annotated upstream of the *IRF2BP1* gene [25]. Their study incorporates nine populations, including East Asian; yet, all of the hand OA subjects were of European or Caucasian ancestries [25].

GWASs have since uncovered ~100 OA genetic risk loci; however, only eight of them are hand OA-related [22,23,24,25,26]. Besides, these findings were primarily replicated in European and Caucasian populations [22,23,24,25,26]. Asian descent remains disproportionately underrepresented in the GWAS of hand OA. Hence, we carried out a GWAS to identify novel genetic susceptibility loci and genes associated with hand OA in 1136 Han Chinese subjects. We also carried out a reporter assay of the candidate SNPs to test their potential functional effects on hand OA.

## 2. Results

### 2.1. Overview of the Study Population

**Figure 1:**
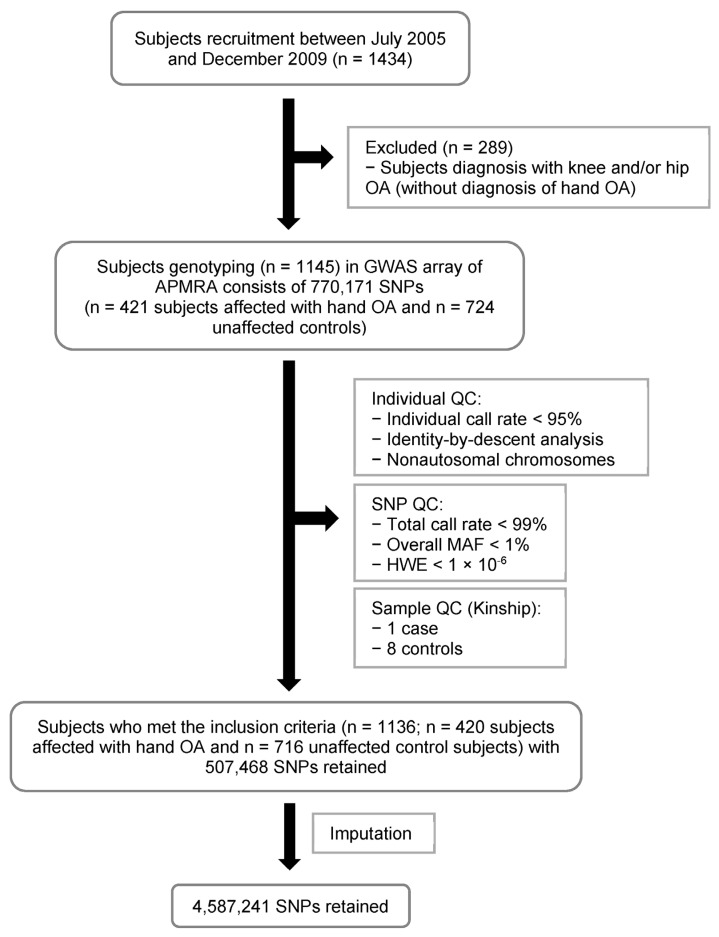
Flowchart of the GWAS hand OA study performed in the Han Chinese population residing in Taiwan.

A total of 1136 unrelated subjects (n = 420 hand OA-affected; n = 716 unaffected control subjects) met the recruitment criteria for this study (Figure 1). Table 1 shows a higher prevalence of female subjects (87%) affected by hand OA than male subjects (13%). Of the unaffected subjects, 76% were female and 24% were male. The mean age was 64.7 and 64.1 years for affected and unaffected subjects, respectively. No significant differences in the BMI between the affected and unaffected control subjects (24.64 vs. 24.67 kg/m^2^). A summary of the sex and age distribution in the GWAS is presented in Supplementary Table S5. The most common age group was 60–69 years for females and 70–79 years for males, in both the affected and unaffected control subjects.

### 2.2. Hand OA Genome-Wide Association Analysis

The MDS plot showed no evidence of population stratification between the GWAS and HapMap population (Supplementary Figure S1A) and between the affected and unaffected control subjects (Supplementary Figure S1B). The genomic inflation factor (λ_GC_) of 1.0418 also revealed a minimal effect of population stratification on the association results. The quantile–quantile (Q–Q) plot is deflated (Supplementary Figure S2), likely due to the moderate sample size.

**Figure 2:**
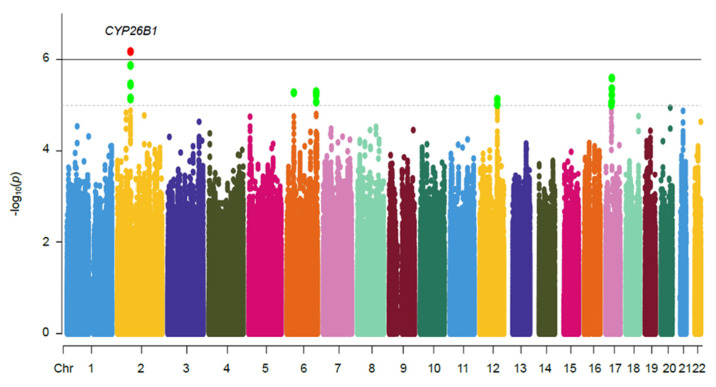
Manhattan plot for hand OA association in Han Chinese population. Manhattan plot of the genome-wide association study of 420 subjects with hand OA and 716 unaffected controls. The *x* axis is each of the SNPs in the initial scan, and the *y* axis is the -log_10_ *p*-value of the Cochran-Armitage trend test. Horizontal dashed lines indicate −log_10_(*p*) = 5, whereas solid black lines indicate −log_10_(*p*) = 6.

**Figure 3:**
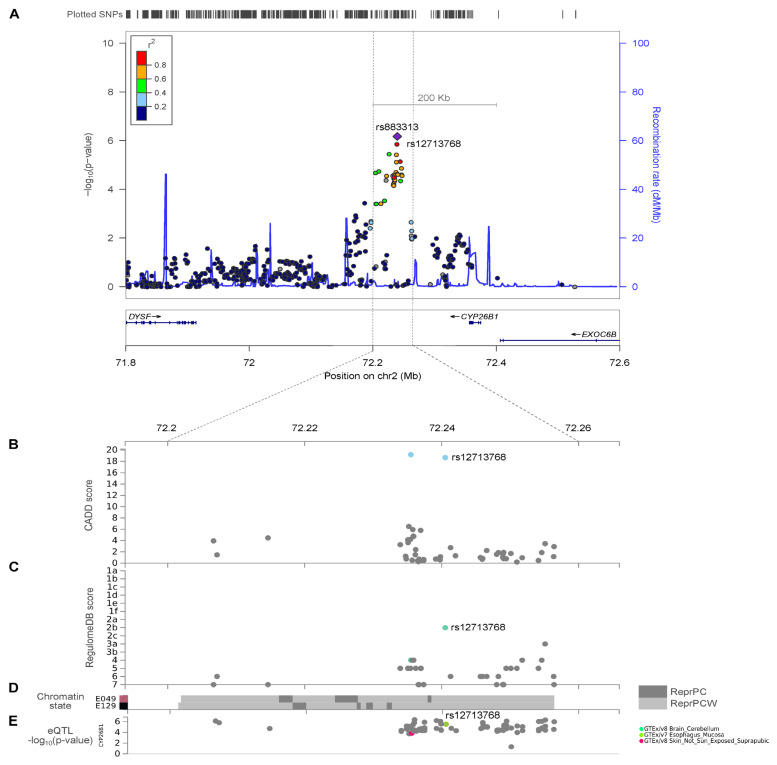
Regional plot for the novel loci associated with hand OA. (**A**) LocusZoom plot of the chromosome 2 locus at SNP rs883313 and rs12713768 showing –log_10_(*p*-value) in the GWAS cohort, recombination rates in Han Chinese study populations, and annotated *CYP26B1* protein coding transcript in the region below. Presented in the zoom in the region surrounding SNP rs12713768 are (**B**) CADD score, (**C**) RegulomeDB score, (**D**) chromatin state data of two tissue types: E049, human mesenchymal stem cell (hMSC)-derived cultured chondrocyte cells and E129, primary osteoblasts, and (**E**) eQTL data of *CYP26B1* generated using FUMA. ReprPC, repressed by Polycomb. ReprPCW, repressed by Polycomb (Weak). eQTL, expression quantitative trait locus.

The Manhattan plot of 4,587,241 SNPs that were tested for the association is shown in Figure 2. The GWAS identified 24 SNPs with a suggestive association (*p*-value < 1 × 10^−5^), of which 21 SNPs were imputed markers (Table 2). The strongest association signal identified was for rs883313 (*p*-value = 6.76 × 10^−7^; OR of 1.76 (95% CI, 1.41–2.19); regional association plot in Figure 3A), followed by rs12713768 (*p*-value = 1.36 × 10^−6^; OR of 1.74 (95% CI, 1.40–2.17)) located downstream of cytochrome P450 family 26 subfamily B member 1 (*CYP26B1*; [MIM]: 605207; Figure 3A). The major C allele of the second strongest SNP rs12713768 is conserved (Supplementary Figure S3A) and is mapped within 150 kb of the *CYP26B1* gene (Supplementary Figure S3B) across 16 mammalian genomes. Three SNPs on chromosome 6 were mapped to the *DNAH8* and *ZRF1PS* genes with a *p*-value of 5.30 × 10^−6^. The other seven SNPs on chromosome 6 were mapped to the *AGPAT4* gene (*p*-value = 5.05–8.50 × 10^−6^). Two markers, rs11108612 and rs11108617, on chromosome 12, were mapped to the *CFAP54* gene (*p*-values = 9.70 × 10^−6^ and 7.45 × 10^−6^, respectively). Two SNPs on chromosome 17 were mapped to the *ASIC2* gene, with *p*-values of 8.90 × 10^−6^ and 9.89 × 10^−6^. The remaining four SNPs on chromosome 17 were mapped to the genes *LYZL6*, *CCL14*, *CCL15*, *CCL16*, and *CCL23,* with *p*-values in the range of 2.56–8.39 × 10^−6^.

### 2.3. CYP26B1/rs12713768 Is a Potential Causal Gene for Hand OA

Subsequent to GWAS, we performed post-GWAS analysis using FUMA to identify causative variants. SNP rs12713768 is predicted to be a potentially deleterious variant, with a C-score of 18.65 (Figure 3B), and it has a regulatory function, with a RegulomeDB rank of 2b (Figure 3C). We also used RegulomeDB to investigate 17 SNPs located on chromosome 2 (mapped within 200 kb to *CYP26B1*) in tissues most relevant to OA, including osteoblasts and chondrocytes. Fifteen of the tested SNPs returned scores of 1-6, four of which were predicted to have regulatory functions, with RegulomeDB scores ≤ 3. Two of these predicted regulatory variants were SNPs with strongest association signal identified in our GWAS (*CYP26B1*/rs883313, RegulomeDB score = 3a; *CYP26B1*/rs12713768, RegulomeDB score = 2b; Supplementary Table S4). Roadmap DHS annotations [27] further showed that SNPs rs12713768 and rs10208040 (LD; D’ = 1, r^2^ = 0.651) were mapped within the enhancer region. This variant is mapped to the polycomb-repressed region in chondrogenic cells and primary osteoblasts (Figure 3D). The C alleles of SNP rs12713768 have also been previously associated with the higher expression of the *CYP26B1* gene according to its eQTL analysis (GTEx Portal database; Figure 3E).

Next, we tested 24 SNPs in an independent hand OA cohort from Geisinger. Data for 20 SNPs were available from Geisinger, and none showed associations with the SNPs of the present study (Supplementary Table S6). We also tested previously reported hand OA-associated SNPs. Some signals are replicated, with *p*-values < 0.05, including 39 SNPs within the *ALDH1A2* [22], SNP rs4764133 near *MGP* gene [23], and SNPs rs7294636 and rs11071366 nearest to *C12orf60* and *ALDH1A2*, respectively [25] (Supplementary Table S7). They did not reach genome-wide significance, but the effects were in the same direction as in our cohort.

To clarify whether our top SNPs rs883313 and rs12713768 are specifically associated with hand OA, we performed GWAS of the 289 subjects affected by knee and/or hip OA (without hand OA). These subjects were previously excluded from our hand OA cohort (Figure 1). These two SNPs, rs883313 and rs12713768, did not reach the suggestive *p*-value threshold of 10^−5^, having *p*-values of 0.3113 and 0.3946, respectively (Supplementary Table S8). In addition, we also estimated the relative risk-conferring allele frequency of these SNPs. SNP rs883313 has a risk allele frequency of 0.766436, while rs12713768 has an allele frequency of 0.768166 compared to the allele frequency of 0.8381 and 0.8405 in the subjects with hand OA, respectively (Supplementary Table S9).

Furthermore, we analyzed the association of SNPs rs883313 and rs12713768 in the Taiwan Precision Medicine Initiative (TPMI) cohorts of hip OA (ICD-10 M16), knee OA (ICD-10 M17), and rheumatoid arthritis (RA; ICD-10 M05 and M06). The *p*-values of these SNPs did not reach *p*-values of <0.05 in all cohorts (Supplementary Table S10). Our findings suggest that these two SNPs are more likely to affect the development and severity of hand OA than those of hip OA, knee OA, and RA.

We further genotyped 33 SNPs in at least 188 hand OA-affected subjects to validate our findings. Fifteen out of the 16 SNPs on chromosome 2 were mapped within 150 kb of the gene *CYP26B1*, and one was within 200 kb. The genotype concordance between the two platforms was ≥98%, except for SNP rs1738263, which had a concordance rate of 96% (Supplementary Table S3). Taken together, our results suggest that rs12713768 is a potential regulatory variant for the novel hand-OA associated gene, *CYP26B1*.

### 2.4. Effect of SNP Variants rs12713768 and rs10208040 on the Predicted Enhancer Activity of CYP26B1 Gene

**Figure 4:**
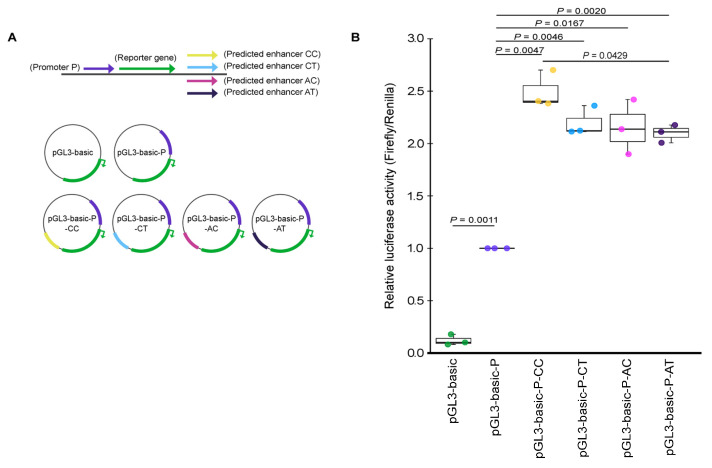
Relative luciferase activity for the human *CYP26B1* promoter and predicted enhancer containing different haplotype of SNPs rs12713768 and rs10208040 in HEK293T cells. (**A**) Different constructs design to evaluate the functionality of promoter and predicted enhancer of different haplotype variants. (**B**) Luciferase activities increased by 2.0-fold with the presence of the predicted enhancer, pGL3-basic-P-CT, pGL3-basic-P-AC, and pGL3-basic-P-AT, as well as by 2.5-fold with the presence of pGL3-basic-P-CC. The presence of risk-conferring CC haplotype within the predicted enhancer (pGL3-basic-P-CC) caused an 18.9% increase in activity (*p*-value = 0.0429) compared with pGL3-basic-P-AT. N = 3 independent experiments. Each dot represents the average of four independent biological replicates of each luciferase assay. Statistical significance changes in reporter gene activity were calculated by the unpaired two-tailed Student’s *t*-test.

**Figure 5:**
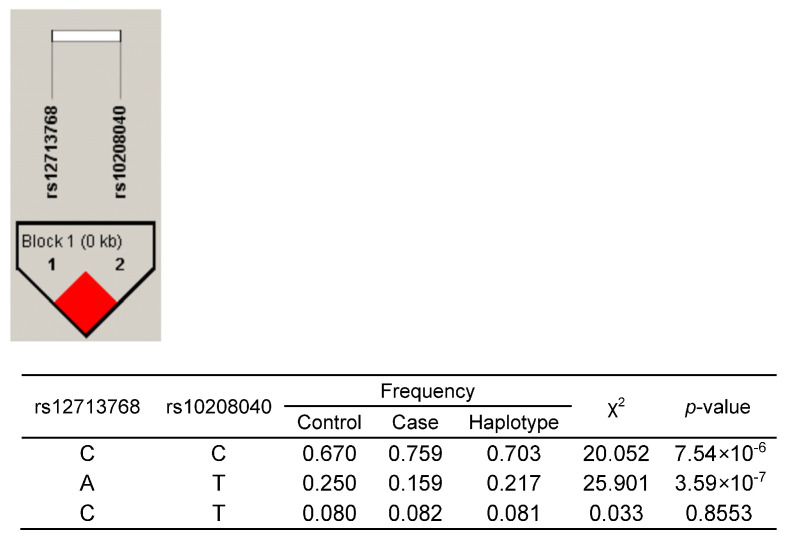
The linkage disequilibrium (LD) pattern (r^2^) of the second strongest SNP signal, rs12713768, generated in Haploview. SNP rs12713768 has strong LD with rs10208040 (D’ = 1, r^2^ = 0.651) in the Han Chinese population.

Two SNPs selected for validation, rs12713768 and rs10208040, were mapped to the predicted enhancer region (2:72,240,035–72,241,404, hg19). We also mapped the promoter of gene *CYP26B1* at the position of 2:72,148,038–72,150,001 (hg19). We performed a reporter assay to determine if the predicted enhancer region is functional and whether SNPs rs12713768 and rs10208040 could affect the regulation of gene expression (Figure 4). Figure 4B shows that the *CYP26B1* promoter has robust activity in driving reporter vector expression as compared to the pGL3-basic vector without a promoter (*p*-value = 0.0011). Alleles A and C of rs12713768 are in strong linkage disequilibrium (LD, D’ = 1, r^2^ = 0.651) with the T and C alleles of rs10208040, respectively (Figure 5). We constructed the promoter and enhancer sequences with AT, AC, CT, and risk-conferring CC haplotype in a pGL3-basic vector (Figure 4A). Sequences with the enhancer showed a 2.0- to 2.5-fold increase in reporter activity compared to the construct with the promoter only, supporting the functionality of the enhancer region (Figure 4B). Moreover, the risk-conferring CC haplotype group displayed 18.9% higher enhancer activity than the AT haplotype group (*p*-value = 0.0429; Figure 4B). This suggests that the presence of risk-conferring CC haplotype in the enhancer region increases reporter gene activity. Our findings confirmed that the predicted enhancer sequence is functional and that the CC haplotype of SNPs rs12713768 and rs10208040 contributed to the increased activity of the enhancer.

## 3. Discussion

We identified novel causal variants, rs883313 and rs12713768, showing the strongest association signals with radiographic hand OA. The risk-conferring CC haplotype of SNPs rs12713768 and rs10208040 (LD, D’ = 1, r^2^ = 0.651), both located downstream nearest to the cytochrome P450 family gene *CYP26B1*, is predicted to increase the expression of *CYP26B1*. Our bioinformatics and functional validation further suggest that *CYP26B1* is potentially a novel causal gene for hand OA.

*CYP26B1* plays an important role in the vitamin A metabolism pathway by catabolizing excess retinoic acid into its inactive polar forms [28]. Retinoic acid, the most active metabolite derivative of vitamin A, is a critical signaling molecule in regulating chondrogenesis and osteogenesis during vertebrate embryonic growth and development [29,30]. Genetic deficiencies in *CYP26B1* in mice display craniofacial abnormalities and limb malformation [31,32]. The *CYP26B1* gene is also highly conserved in all chordates, further affirming its critical role in vertebrate development [33].

A retinoic acid-related gene (*ALDH1A2*) had previously been linked to the hand OA phenotype in the Icelandic population [22]. The *ALDH1A2* gene encodes an enzyme involved in retinoic acid synthesis [22]. Studies by Styrkarsdottir et al. [22] and Shepherd et al. [34] revealed that the reduced bioavailability of retinoic acid might increase the risk of hand OA. Higher expression of *CYP26B1* in OA hip cartilage compared to non-OA control hip cartilage was also observed in their RNA-seq data, albeit it was not significantly differentially expressed [34]. On the contrary, Davies et al. [35] proposed that excess levels of retinoic acid detected in synovial fluid of OA patients may cause detrimental effects on the cartilage. Taking our findings into consideration, we deduce that increased expression of *CYP26B1* may also lower the level of retinoic acid in the tissues and lead to the same phenotypic outcome as Styrkarsdottir et al. [22] and Shepherd et al. [34]. The low bioavailability of retinoic acid is known to compromise cartilage integrity through modulation of the expression of a key chondrogenic transcription factor, SRY-Box Transcription Factor 9 (*SOX9*) [34]. Our study highlights the importance of retinoic acid and vitamin A metabolism pathway for hand OA phenotype in both European and Han Chinese populations, despite affecting different genes.

Joint pain is one of the hallmark symptoms of OA, and the treatment of OA is mainly aimed at pain relief [36]. Our GWAS identified several genes that have been documented for their functional relationship with OA pain (*AGPAT4* [37] and *ASIC2* [38,39,40]) and low bone mineral content (*AGPAT4* [41]). *AGPAT4* (1-acylglycerol-3-phosphate O-acyltransferase 4) encodes a catalyze enzyme involved in the conversion of lysophosphatic acid (LPA) to phosphatidic acid in the phospholipid biosynthesis [41]. McDougall et al. [37] has demonstrated that LPA contributes to the neuropathic component of OA. Furthermore, *AGPAT4*-knockout mice showed a decrease in bone mineral content [41]. *ASIC2* (acid-sensing ion channel 2) is a member of the proton-gated cation channels [42]. It is a nociceptor that is part of the amiloride-sensitive degenerin/epithelial sodium channel (DEG/ENaC) superfamily [39,42]. Previous studies demonstrated that *ASIC2* expressed in bone cells may have a role in regulating the pain signal transmission associated with bone metabolism [39,40]. Cho et al. [38] observed an increase in the mRNA expression and protein levels of *ASIC2* in the frozen shoulder patients as compared to the controls, implying its possible role in the pathogenesis of shoulder pain caused by frozen shoulder.

We also identified several genes expressing inflammatory chemokines that previously have been detected in the inflamed synovium samples of OA patients (*CCL14* [43], *CCL15* [43], and *CCL16* [43]) or have been reported to be expressed in human fetal osteoblast and chondrocytes (*CCL23* [44]). *CCL15* was also detected to significantly elevate in the severe-stage OA group compared to the early-stage OA group [45]. The involvement of inflammatory chemokine in the pathogenesis of OA has gained more attention, as it has been known that chemokine can be secreted by chondrocytes and synovial cells, which can be detected in the synovial fluid of OA patients [45,46,47]. However, the complexity of the inflammation-induced signaling pathway warrants more studies to define the mechanisms in which chemokine may be involved in the pathogenesis of OA.

Nevertheless, the top SNPs identified in this study were not replicated in an independent cohort from Geisinger. These differences are most likely attributed to ethnic heterogeneity, as more than 95% of the Geisinger population is of white European ancestry [48]. SNP rs12713768 has a risk allele frequency of 47% and 55% in American and European populations, respectively, but it is higher, at 77%, in the East Asian population, according to the 1000 Genome Project (Phase 3; Supplementary Table S11). In addition, the Geisinger cohort used the ICD codes, rather than the strict radiographic criteria used in this study, which could also contribute to the differences in results.

We were also not able to validate the previously reported hand OA-associated variants. This could be owing to the relatively small sample size used in this study, heterogeneity of hand OA phenotypes and ethnic groups, and different case definitions (radiographically confirmed cases of hand OA in the present study as compared to ICD codes). The undetermined misclassification rate was a weakness for some of the previous studies and the Geisinger cohort used in this study. On the other hand, the lack of replication suggests the complexity of the polygenic background of hand OA, which involves multiple genes, variable phenotypic penetrance of the variants, and complex gene–environment interactions.

For the first time, our study revealed the association between *CYP26B1* gene with hand OA. We identified SNPs located within a functional enhancer region mapped closest to the vitamin A metabolizing gene *CYP26B1*, which likely accounts for the progression of hand OA through the reduced bioavailability of retinoic acid. Future studies with larger sample sizes are required to replicate these findings and to identify markers that are weakly associated with the target traits. In addition, we also identified several genes linked to joint pain and inflammation that have not been associated with hand OA. However, further analysis is required to demonstrate their potential role as candidate genes for hand OA or to delineate their molecular mechanism in the association with hand OA. Our findings, together with previous genetic studies [22,34], highlight the genetic contribution of target SNP variants in the vitamin A metabolism pathway in association with the severity of hand OA.

## 4. Methods

### 4.1. Study Populations

A total of 1434 subjects were recruited from five medical centers in Taiwan (National Taiwan University Hospital, Taipei Medical University Hospital, Taipei Veterans General Hospital, Tri-Service General Hospital, and Linkou Chang Gung Memorial Hospital) between 2005 and 2009 (Figure 1). All hand OA-affected and unaffected control subjects were of Taiwanese Han background, and range in age from 32 to 94 years (with a mean of 64.4 years; Table 1).

### 4.2. Phenotype Definition for Hand OA

#### 4.2.1. Inclusion Criteria

The inclusion criteria to define hand OA cases follow those of The Genetics of Generalized Osteoarthritis (GOGO) study [49]. The severity of hand OA was graded according to the Kellgren-Lawrence (KL) scaling system (grades 0–4) [50]. Radiographic hand OA was defined as the presence of three or more joint involvements of KL grade ≥ 2, at least one DIP of digits 2–5, two of the three involved joints within a joint group (DIP, PIP, or CMC), with the first IP joint of a thumb being considered in the PIP group, bilateral hand involvement, and no more than three swollen metacarpophalangeal (MCP) joints ≥2 by clinical examination, as defined in the Dictionary of Rheumatic Diseases [51].

#### 4.2.2. Exclusion Criteria

Subjects were excluded if they had a diagnosis of bone and joint disease (including rheumatoid arthritis, gout arthritis, psoriatic arthropathy, hypertrophic osteoarthropathy, hypermotility syndrome, hemochromatosis, Paget’s disease, spondyloarthropathy, post-traumatic OA, other secondary-form OA, or lupus) or had more than three swollen MCP joints with KL grade ≥ 2, or they were excluded if they had MCP changes compatible with hemochromatosis. Additionally, if fasting transferrin saturation (FE/TIBC) ratio outcomes were >55%, the subjects were excluded. Female subjects who had a positive urine pregnancy test at screening were excluded.

### 4.3. Phenotype Definition of Non-OA Control Subjects

#### 4.3.1. Inclusion Criteria

Non-OA controls for the study were recruited of age and ethnic match to the hand OA-affected subjects. All non-OA control subjects were age ≥ 30 years of Taiwanese Han ancestry. Non-OA controls were defined as subjects without OA in hands, hips, and knees (no more than two hand joints involvement of KL grade ≥ 2, hip-KL grade of <2, and knee-KL grade < 2, respectively). If greater than three joints with KL grade ≥ 2 were present in hand joints, then it should be neither bilateral joint involvement nor two joints in the same joint group (DIP, PIP or CMC). If the hip-KL grade is equal to 2, the review joint space width has to be ≥2.5.

#### 4.3.2. Exclusion Criteria

Subjects with OA in the hand joint, or had hip and/or knee OA, or had any bone and joint disease as mentioned above, were excluded. Subjects with a knee-KL grade ≥ 2, and/or a hip-KL grade ≥ 2, or those that had any osteophytes in the hip with joint space width < 2.5, were excluded. Subjects were excluded if they had more than three swollen MCP joints with KL grade ≥ 2, MCP changes compatible with hemochromatosis, or iron overload > 55%. None of the first-degree relative subjects was enrolled in the study.

Of the 1434 subjects who participated, 289 individuals having knee and/or hip OA (without hand OA) were excluded from the analysis (Figure 1). A total of 1145 individuals were genotyped (n = 421 hand OA-affected; n = 724 unaffected control subjects). The replication cohort was identified from within the Geisinger MyCode community health initiative (MyCode^®^), a system-wide research biorepository in Geisinger, Pennsylvania, USA with more than 265,000 participants enrolled to date [48]. Hand OA-affected and control population of the Geisinger cohort was identified using International Classification of Diseases (ICD) codes (Supplementary Table S1).

### 4.4. Genotyping, Quality Control, and Imputation

Genomic DNA was extracted from the blood using the QIAamp DNA Blood Mini kit (QIAGEN Inc., Valencia, CA, USA). Genotyping was performed using the Axiom Asia Precision Medicine Research Array (Affymetrix, Santa Clara, CA, USA). Genotype calls were determined using the BirdSeed genotyping algorithm implemented in Affymetrix Power Tools (Affymetrix, Santa Clara, CA, USA). The overall call rates of all samples were >95%. Per-individual quality control (QC) excluded the following samples for further analysis: (i) call rate < 95%; (ii) discrepancy between recorded and genotype-determined sex; (iii) first-degree relatives identified by kinship analysis; and (iv) outliers in the multidimensional scaling plot. Genotype QC for each SNP was evaluated by the total call rate and minor allele frequency (MAF) in cases and controls combined. Autosomal SNPs that met the following criteria were excluded from further analysis: (i) nonpolymorphic, (ii) sample call rate < 95%, (iii) MAF < 1% and total call rate < 99%, and (iv) Hardy-Weinberg equilibrium *p*-value < 1 × 10^−6^. After QC and removing individuals who had kinship relationships (one in cases and eight in controls), 507,468 SNPs were retained in the 420 hand OA-affected and 716 unaffected control subjects (Supplementary Table S2). Genotype data passed QC were imputed with IMPUTE2 (https://mathgen.stats.ox.ac.uk/impute/impute_v2.html) with East Asian 1000 Genomes Project data as the references. After imputation (info score > 0.9), 4,587,241 SNPs were obtained.

### 4.5. Genome-Wide SNPs Cross-Platform Validation

Thirty-three SNPs that are located nearest to *CYP26B1* or have *p*-values < 1 × 10^−5^ were further validated (Supplementary Table S3). Top SNPs rs883313 and rs12713768 were validated in all controls and cases (n = 1136). The other 31 SNPs were validated in at least 188 hand OA-affected subjects in the current study. SNPs of interest were genotyped using either the Sequenom MassARRAY iPLEX platform (Sequenom, San Diego, CA, USA) at the National Center for Genome Medicine, Academia Sinica, Taiwan, or standard Sanger direct sequencing on an ABI Prism 3730XL DNA Analyser (Applied Biosystems, Foster, CA, USA).

### 4.6. Statistical and Bioinformatics Database Analysis

All statistical analyses were performed using PLINK1.07 (http://pngu.mgh.harvard.edu/~purcell/plink). Multidimensional scaling (MDS) showed that all individuals included in the study were clustered closely within the East Asian 1000 Genomes Project. The genomic variance inflation factor (λ_GC_), calculated according to Devlin et al. [52], was 1.0418. We performed the Cochran-Armitage trend test to compare allele and genotype frequencies between cases and controls. The distribution of the *p*-value was examined by plotting a quantile–quantile (Q–Q) plot. Logistic regression models were performed to evaluate the odds ratio (OR) with 95% confidence intervals (CI). Manhattan and Q–Q plots were generated using the CMplot package (https://CRAN.R-project.org/package=CMplot; accessed on 12 March 2021) [53]. The locus-specific plot was generated using LocusZoom (http://locuszoom.sph.umich.edu/locuszoom/), with recombination rates taken from the East Asian component of the 1000 Genomes Project. Haplotype analysis was performed using SNPStats [54] and Haploview [55].

Variant regulatory region annotation was performed using the SNP2GENE tool from Functional Mapping and Annotation (FUMA) [56]. FUMA annotation includes combined annotation-dependent depletion (CADD) score [57], RegulomeDB score [58], chromatin state, and expression quantitative trait loci (eQTL) data. CADD ranks the pathogenicity of a variant as a C-score ranging from 1 to 99. Any variant with a C-score ≥ 10 is considered to be within the top 10% of deleteriousness substitutions in the human genome [59]. RegulomeDB online database (https://regulomedb.org/; accessed on 27 August 2021) [58] was used to explore the functionality of 17 SNPs on chromosome 2 in tissues most relevant to OA (Supplementary Table S4). It scores SNP functionality according to experimental datasets from the Encyclopedia of DNA Elements (ENCODE) project, Gene Expression Omnibus, and the published literature covering 962 datasets and over 100 tissues and cell lines [58]. SNPs are graded from ranks 1-6, with lower rank indicating the more likely the SNP is to be located within a potentially functional region [58].

### 4.7. Construction of Luciferase Reporter Plasmids

SNPs rs12713768 and rs10208040 (strong linkage disequilibrium (LD); D’ = 1, r^2^ = 0.651) were further selected for functional validation, as they were mapped to the predicted enhancer region (2:72,240,035–72,241,404, hg19) according to Roadmap DNase hypersensitivity site (DHS) annotations [27]. Five constructs were synthesized and cloned into the pGL3-basic vector by Bio Basic Inc. (Markham, ON, Canada). The pGL3-basic plasmid was digested with *Sac*I and *Mlu*I, where the DNA fragment of chromosome 2:72,148,038–72,150,001 (hg19) was ligated to generate the pGL3-basic-P construct. The DNA fragment was excerpted from the promoter region of *CYP26B1* (contains the region of ENSR00000118913 but without the region ENSE00001956510) according to Ensembl Database (http://www.ensembl.org/; accessed on 7 August 2020). Four of the pGL3-basic-P constructs were digested with *Bam*HI and *Sal*I. The predicted enhancer sequences with different interest variants of rs12713768 (2:72,240,527, hg19; C or A allele) and rs10208040 (2:72,241,295, hg19; C or T allele) were ligated into these constructs, generating constructs with risk haplotype, pGL3-basic-P-CC, pGL3-basic-P-CT, pGL3-basic-P-AC, and construct with alternative haplotype, pGL3-basic-P-AT. Positive clones were sequence-verified by Sanger sequencing. *Renilla* luciferase, the pRL-TK vector, was used as an internal control, while pGL3-basic vector was used as a negative control. Plasmid DNA was isolated using PureLink™ HiPure Plasmid Maxiprep Kit (Invitrogen, Waltham, MA, USA).

### 4.8. Cell Cultures and Luciferase Reporter Assay

Human embryonic kidney (HEK) 293T cells were maintained at 37 °C and 5% CO_2_ in Dulbecco’s modified essential medium, containing 10% FBS, 100 U/mL penicillin, and 100 μg/mL streptomycin antibiotics. Liposome-based transfection was carried out using Lipofectamine 2000 (Invitrogen, Waltham, MA, USA) according to the manufacturer’s instructions. HEK293T cells were seeded into 96-well plates at a density of 10,000 cells per well for 24 h. Cells were co-transfected with the same plasmid copy number of each construct DNA and pRL-TK and lysed after 24 h. Luminescence was measured using Firefly & *Renilla* Luciferase Single Tube Assay Kit (Biotium, Fremont, CA, USA) on an EnSpire™ Multilabel Plate Reader (Perkin Elmer, Waltham, MA, USA). Firefly luciferase activity was normalized to the activity of *Renilla* luciferase. Three independent experiments, with at least four technical replicates, were performed per construct. The relative luciferase activities are presented regarding the activity of pGL3-basic-P construct being defined as 1.

### 4.9. Statistics for Luciferase Assays

Statistical analysis was conducted in R 4.1.1 (https://www.R-project.org/), and a *p*-value < 0.05 was considered significant. *p*-values were calculated using the unpaired two-tailed Student’s *t*-test. Assay figures were plotted using the package ggplot2 [60].

## Figures and Tables

**Table 1:** Basic characteristics of study populations.

Characteristic	Affected	Unaffected Control
N	420	716
F/M (n, %)	365 (87)/55 (13)	547 (76)/169 (24)
Age at sampling, years ± SD ^a^ (min–max)	64.7 ± 10.4 (32–94)	64.1 ± 9.7 (34–93)
Height, cm ± SD ^b^ (min–max)	155.81 ± 6.9 (137–184)	157.58 ± 6.9 (138.5–180)
Weight, kg ± SD (min–max)	59.87 ± 9.21 (36–100)	61.32 ± 9.91 (35–106)
BMI, kg/m^2^ ± SD ^b^ (min–max)	24.64 ± 3.34 (15.51–39.11)	24.67 ± 3.53 (13.01–36.16)
BMI ≤ 24.9 kg/m^2^	244 (58.10%)	414 (57.90%)
25.0 > BMI < 29.9 kg/m^2^	155 (36.90%)	246 (34.41%)
BMI ≥ 30.9 kg/m^2^	21 (5%)	55 (7.69%)

BMI, body mass index. ^a^ mean ± standard deviation; ^b^ Data on height was missing for one unaffected control subject.

**Table 2:** Top SNPs associated with hand OA in the GWAS.

Chr	SNP	Position (bp)	Allele Format	Risk Allele	Risk Allele OR (95% CI)	RAF Controls	RAF Cases	*p*-Value	Nearest Gene ^a^	
2	rs1517396	72,228,008	CT	T	1.588 (1.308–1.928)	0.6568	0.7525	3.43 × 10^−6^	*CYP26B1*	Imputed
2	rs883313	72,239,692	TC	C	1.755 (1.409–2.185)	0.7468	0.8381	6.76 × 10^−7^	*CYP26B1*	Imputed
2	rs883312	72,239,762	CT	T	1.581 (1.305–1.916)	0.6620	0.7560	3.65 × 10^−6^	*CYP26B1*	
2	rs883311	72,239,809	GA	A	1.560 (1.287–1.891)	0.6636	0.7548	7.31 × 10^−6^	*CYP26B1*	Imputed
2	rs12713768	72,240,527	AC	C	1.737 (1.393–2.164)	0.7521	0.8405	1.36 × 10^−6^	*CYP26B1*	
2	rs191066740	72,245,915	TG	G	1.721 (1.364–2.173)	0.7746	0.8554	6.97 × 10^−6^	*CYP26B1*	Imputed
6	rs1614699	38,738,748	AT	A	1.809 (1.399–2.338)	0.0949	0.1595	5.30 × 10^−6^	*DNAH8, ZRF1PS*	Imputed
6	rs1678657	38,742,793	GT	G	1.809 (1.399–2.338)	0.0949	0.1595	5.30 × 10^−6^	*DNAH8, ZRF1PS*	Imputed
6	rs1738263	38,743,252	CT	C	1.809 (1.399–2.338)	0.0949	0.1595	5.30 × 10^−6^	*DNAH8, ZRF1PS*	Imputed
6	rs12197517	161,666,190	CG	G	2.327 (1.577–3.432)	0.9088	0.9586	8.50 × 10^−6^	*AGPAT4*	Imputed
6	rs62437572	161,666,765	CT	T	2.360 (1.601–3.479)	0.9076	0.9586	5.62 × 10^−6^	*AGPAT4*	Imputed
6	rs10945720	161,668,745	AG	G	2.366 (1.605–3.488)	0.9076	0.9587	5.25 × 10^−6^	*AGPAT4*	Imputed
6	rs12190239	161,669,231	GC	C	2.362 (1.602–3.482)	0.9077	0.9587	5.48 × 10^−6^	*AGPAT4*	Imputed
6	rs112790908	161,670,764	AAT	AT	2.358 (1.600–3.477)	0.9079	0.9587	5.72 × 10^−6^	*AGPAT4*	Imputed
6	rs12207205	161,671,403	AG	G	2.367 (1.606–3.489)	0.9075	0.9587	5.05 × 10^−6^	*AGPAT4*	Imputed
6	rs73019329	161,678,741	CT	T	2.353 (1.596–3.469)	0.9083	0.9588	6.06 × 10^−6^	*AGPAT4*	Imputed
12	rs11108612	97,041,461	AG	G	1.484 (1.244–1.771)	0.5380	0.6335	9.70 × 10^−6^	*CFAP54*	Imputed
12	rs11108617	97,047,776	AG	G	1.499 (1.253–1.792)	0.5359	0.6338	7.45 × 10^−6^	*CFAP54*	Imputed
17	rs76055737	31,565,197	TC	C	1.947 (1.442–2.629)	0.8632	0.9248	9.89 × 10^−6^	*ASIC2*	Imputed
17	rs79186365	31,565,269	AG	G	1.953 (1.447–2.638)	0.8629	0.9248	8.90 × 10^−6^	*ASIC2*	Imputed
17	rs111761296	34,309,769	CCCCTT	C	2.124 (1.552–2.905)	0.0579	0.1156	2.56 × 10^−6^	*LYZL6, CCL14, CCL15, CCL16, CCL23*	Imputed
17	rs76190126	34,310,256	AC	A	2.024 (1.485–2.758)	0.0601	0.1147	8.39 × 10^−6^	*LYZL6, CCL14, CCL15, CCL16, CCL23*	Imputed
17	rs10491118	34,314,886	TG	T	1.971 (1.475–2.636)	0.0677	0.1253	5.99 × 10^−6^	*LYZL6, CCL14, CCL15, CCL16, CCL23*	Imputed
17	rs12051658	35,988,597	GA	G	1.988 (1.488–2.656)	0.0677	0.1262	4.35 × 10^−6^	*LYZL6, CCL14, CCL15, CCL16, CCL23*	

Results are shown for SNPs with *p* < 1 × 10^−5^. Chr, chromosome; OR, odds ratio for risk allele; 95% CI, 95% confidence interval; RAF, risk allele frequency; *p*-value, trend *p*-value of Cochran-Armitage test; Nearest gene, gene nearest to the SNPs; ^a^ Nearest gene is within 150 kb away.

## Data Availability

The data supporting the findings of this study are available within the article or its Supplementary Materials.

## Supplementary Materials

The following supporting information can be downloaded at: https://www.mdpi.com/article/10.3390/ijms24033021/s1.

## References

[1] Kloppenburg M., Kwok W.-Y. (2011). Hand osteoarthritis—A heterogeneous disorder. Nat. Rev. Rheumatol..

[2] Marshall M., Watt F.E., Vincent T.L., Dziedzic K. (2018). Hand osteoarthritis: Clinical phenotypes, molecular mechanisms and disease management. Nat. Rev. Rheumatol..

[3] Leung G.J., Rainsford K.D., Kean W.F. (2014). Osteoarthritis of the hand I: Aetiology and pathogenesis, risk factors, investigation and diagnosis. J. Pharm. Pharmacol..

[4] van Saase J.L., van Romunde L.K., Cats A., Vandenbroucke J.P., Valkenburg H.A. (1989). Epidemiology of osteoarthritis: Zoetermeer survey. Comparison of radiological osteoarthritis in a Dutch population with that in 10 other populations. Ann. Rheum. Dis..

[5] Carmona L., Ballina J., Gabriel R., Laffon A., EPISER Study Group (2001). The burden of musculoskeletal diseases in the general population of Spain: Results from a national survey. Ann. Rheum. Dis..

[6] Zhang Y., Xu L., Nevitt M.C., Niu J., Goggins J.P., Aliabadi P., Yu W., Lui L.-Y., Felson D.T. (2003). Lower prevalence of hand osteoarthritis among Chinese subjects in Beijing compared with white subjects in the United States: The Beijing Osteoarthritis Study. Arthritis Rheum..

[7] Kodama R., Muraki S., Oka H., Iidaka T., Teraguchi M., Kagotani R., Asai Y., Yoshida M., Morizaki Y., Tanaka S. (2016). Prevalence of hand osteoarthritis and its relationship to hand pain and grip strength in Japan: The third survey of the ROAD study. Mod. Rheumatol..

[8] Sowers M., Lachance L., Hochberg M., Jamadar D. (2000). Radiographically defined osteoarthritis of the hand and knee in young and middle-aged African American and Caucasian women. Osteoarthr. Cartil..

[9] Toba N., Sakai A., Aoyagi K., Yoshida S., Honda S., Nakamura T. (2006). Prevalence and involvement patterns of radiographic hand osteoarthritis in Japanese women: The Hizen-Oshima Study. J. Bone Miner. Metab..

[10] Bang S.-Y., Son C.-N., Sung Y.-K., Choi B.K., Joo K.-B., Jun J.-B. (2011). Joint-specific prevalence and radiographic pattern of hand osteoarthritis in Korean. Rheumatol. Int..

[11] Zhang R., Yao J., Xu P., Ji B., Luck J.V., Chin B., Lu S., Kelsoe J.R., Ma J. (2015). A comprehensive meta-analysis of association between genetic variants of GDF5 and osteoarthritis of the knee, hip and hand. Inflamm. Res..

[12] Chapman K., Takahashi A., Meulenbelt I., Watson C., Rodriguez-Lopez J., Egli R., Tsezou A., Malizos K.N., Kloppenburg M., Shi D. (2008). A meta-analysis of European and Asian cohorts reveals a global role of a functional SNP in the 5’ UTR of GDF5 with osteoarthritis susceptibility. Hum. Mol. Genet..

[13] Miyamoto Y., Mabuchi A., Shi D., Kubo T., Takatori Y., Saito S., Fujioka M., Sudo A., Uchida A., Yamamoto S. (2007). A functional polymorphism in the 5’ UTR of GDF5 is associated with susceptibility to osteoarthritis. Nat. Genet..

[14] Stecher R.M. (1940). Heberden’s nodes: The incidence of hypertrophic arthritis of the fingers. N. Engl. J. Med..

[15] Spector T.D., Cicuttini F., Baker J., Loughlin J., Hart D. (1996). Genetic influences on osteoarthritis in women: A twin study. BMJ.

[16] Horton W.E., Lethbridge-Cejku M., Hochberg M.C., Balakir R., Precht P., Plato C.C., Tobin J.D., Meek L., Doege K. (1998). An association between an aggrecan polymorphic allele and bilateral hand osteoarthritis in elderly white men: Data from the Baltimore Longitudinal Study of Aging (BLSA). Osteoarthr. Cartil..

[17] Kirk K.M., Doege K.J., Hecht J., Bellamy N., Martin N.G. (2003). Osteoarthritis of the hands, hips and knees in an Australian twin sample--evidence of association with the aggrecan VNTR polymorphism. Twin Res..

[18] Kämäräinen O.P., Solovieva S., Vehmas T., Luoma K., Leino-Arjas P., Riihimäki H., Ala-Kokko L., Männikkö M. (2006). Aggrecan core protein of a certain length is protective against hand osteoarthritis. Osteoarthr. Cartil..

[19] Ross J.M., Kowalchuk R.M., Shaulinsky J., Ross L., Ryan D., Phatak P.D. (2003). Association of heterozygous hemochromatosis C282Y gene mutation with hand osteoarthritis. J. Rheumatol..

[20] Carroll G.J. (2006). HFE gene mutations are associated with osteoarthritis in the index or middle finger metacarpophalangeal joints. J. Rheumatol..

[21] Zhai G., van Meurs J.B.J., Livshits G., Meulenbelt I., Valdes A.M., Soranzo N., Hart D., Zhang F., Kato B.S., Richards J.B. (2009). A genome-wide association study suggests that a locus within the ataxin 2 binding protein 1 gene is associated with hand osteoarthritis: The Treat-OA consortium. J. Med. Genet..

[22] Styrkarsdottir U., Thorleifsson G., Helgadottir H.T., Bomer N., Metrustry S., Bierma-Zeinstra S., Strijbosch A.M., Evangelou E., Hart D., Beekman M. (2014). Severe osteoarthritis of the hand associates with common variants within the ALDH1A2 gene and with rare variants at 1p31. Nat. Genet..

[23] den Hollander W., Boer C.G., Hart D.J., Yau M.S., Ramos Y.F.M., Metrustry S., Broer L., Deelen J., Cupples L.A., Rivadeneira F. (2017). Genome-wide association and functional studies identify a role for matrix Gla protein in osteoarthritis of the hand. Ann. Rheum. Dis..

[24] Boer C.G., Yau M.S., Rice S.J., Coutinho de Almeida R., Cheung K., Styrkarsdottir U., Southam L., Broer L., Wilkinson J.M., Uitterlinden A.G. (2020). Genome-wide association of phenotypes based on clustering patterns of hand osteoarthritis identify WNT9A as novel osteoarthritis gene. Ann. Rheum. Dis..

[25] Boer C.G., Hatzikotoulas K., Southam L., Stefánsdóttir L., Zhang Y., Coutinho de Almeida R., Wu T.T., Zheng J., Hartley A., Teder-Laving M. (2021). Deciphering osteoarthritis genetics across 826,690 individuals from 9 populations. Cell.

[26] Reynard L.N., Barter M.J. (2020). Osteoarthritis year in review 2019: Genetics, genomics and epigenetics. Osteoarthr. Cartil..

[27] Liu Y., Chang J.-C., Hon C.-C., Fukui N., Tanaka N., Zhang Z., Lee M.T.M., Minoda A. (2018). Chromatin accessibility landscape of articular knee cartilage reveals aberrant enhancer regulation in osteoarthritis. Sci. Rep..

[28] White J.A., Ramshaw H., Taimi M., Stangle W., Zhang A., Everingham S., Creighton S., Tam S.P., Jones G., Petkovich M. (2000). Identification of the human cytochrome P450, P450RAI-2, which is predominantly expressed in the adult cerebellum and is responsible for all-trans-retinoic acid metabolism. Proc. Natl. Acad. Sci. USA.

[29] MacLean G., Abu-Abed S., Dollé P., Tahayato A., Chambon P., Petkovich M. (2001). Cloning of a novel retinoic-acid metabolizing cytochrome P450, Cyp26B1, and comparative expression analysis with Cyp26A1 during early murine development. Mech. Dev..

[30] Williams J.A., Kane M., Okabe T., Enomoto-Iwamoto M., Napoli J.L., Pacifici M., Iwamoto M. (2010). Endogenous retinoids in mammalian growth plate cartilage: Analysis and roles in matrix homeostasis and turnover. J. Biol. Chem..

[31] Yashiro K., Zhao X., Uehara M., Yamashita K., Nishijima M., Nishino J., Saijoh Y., Sakai Y., Hamada H. (2004). Regulation of retinoic acid distribution is required for proximodistal patterning and outgrowth of the developing mouse limb. Dev. Cell.

[32] Maclean G., Dollé P., Petkovich M. (2009). Genetic disruption of CYP26B1 severely affects development of neural crest derived head structures, but does not compromise hindbrain patterning. Dev. Dyn..

[33] Carvalho J.E., Theodosiou M., Chen J., Chevret P., Alvarez S., De Lera A.R., Laudet V., Croce J.C., Schubert M. (2017). Lineage-specific duplication of amphioxus retinoic acid degrading enzymes (CYP26) resulted in sub-functionalization of patterning and homeostatic roles. BMC Evol. Biol..

[34] Shepherd C., Zhu D., Skelton A.J., Combe J., Threadgold H., Zhu L., Vincent T.L., Stuart P., Reynard L.N., Loughlin J. (2018). Functional Characterization of the Osteoarthritis Genetic Risk Residing at ALDH1A2 Identifies rs12915901 as a Key Target Variant. Arthritis Rheumatol..

[35] Davies M.R., Ribeiro L.R., Downey-Jones M., Needham M.R.C., Oakley C., Wardale J. (2009). Ligands for retinoic acid receptors are elevated in osteoarthritis and may contribute to pathologic processes in the osteoarthritic joint. Arthritis Rheum..

[36] Schaefer L.F., McAlindon T.E., Eaton C.B., Roberts M.B., Haugen I.K., Smith S.E., Duryea J., Driban J.B. (2018). The associations between radiographic hand osteoarthritis definitions and hand pain: Data from the osteoarthritis initiative. Rheumatol. Int..

[37] McDougall J.J., Albacete S., Schuelert N., Mitchell P.G., Lin C., Oskins J.L., Bui H.H., Chambers M.G. (2017). Lysophosphatidic acid provides a missing link between osteoarthritis and joint neuropathic pain. Osteoarthr. Cartil..

[38] Cho C.H., Lho Y.M., Ha E., Hwang I., Song K.S., Min B.W., Bae K.C., Kim D.H. (2015). Up-regulation of acid-sensing ion channels in the capsule of the joint in frozen shoulder. Bone Jt. J..

[39] Kanaya K., Iba K., Dohke T., Okazaki S., Yamashita T. (2016). TRPV1, ASICs and P2X2/3 expressed in bone cells simultaneously regulate bone metabolic markers in ovariectomized mice. J. Musculoskelet. Neuronal Interact..

[40] Hanaka M., Iba K., Dohke T., Kanaya K., Okazaki S., Yamashita T. (2018). Antagonists to TRPV1, ASICs and P2X have a potential role to prevent the triggering of regional bone metabolic disorder and pain-like behavior in tail-suspended mice. Bone.

[41] Suzuki A., Minamide M., Iwaya C., Ogata K., Iwata J. (2020). Role of metabolism in bone development and homeostasis. Int. J. Mol. Sci..

[42] Jahr H., van Driel M., van Osch G.J.V.M., Weinans H., van Leeuwen J.P.T.M. (2005). Identification of acid-sensing ion channels in bone. Biochem. Biophys. Res. Commun..

[43] Haringman J.J., Smeets T.J.M., Reinders-Blankert P., Tak P.P. (2006). Chemokine and chemokine receptor expression in paired peripheral blood mononuclear cells and synovial tissue of patients with rheumatoid arthritis, osteoarthritis, and reactive arthritis. Ann. Rheum. Dis..

[44] Votta B.J., White J.R., Dodds R.A., James I.E., Connor J.R., Lee-Rykaczewski E., Eichman C.F., Kumar S., Lark M.W., Gowen M. (2000). CKbeta-8 [CCL23], a novel CC chemokine, is chemotactic for human osteoclast precursors and is expressed in bone tissues. J. Cell Physiol..

[45] Gao K., Zhu W., Li H., Ma D., Liu W., Yu W., Wang L., Cao Y., Jiang Y. (2020). Association between cytokines and exosomes in synovial fluid of individuals with knee osteoarthritis. Mod. Rheumatol..

[46] Vergunst C.E., van de Sande M.G.H., Lebre M.C., Tak P.P. (2005). The role of chemokines in rheumatoid arthritis and osteoarthritis. Scand. J. Rheumatol..

[47] Goldring M.B., Otero M. (2011). Inflammation in osteoarthritis. Curr. Opin. Rheumatol..

[48] Carey D.J., Fetterolf S.N., Davis F.D., Faucett W.A., Kirchner H.L., Mirshahi U., Murray M.F., Smelser D.T., Gerhard G.S., Ledbetter D.H. (2016). The Geisinger MyCode community health initiative: An electronic health record-linked biobank for precision medicine research. Genet. Med..

[49] Kraus V.B., Jordan J.M., Doherty M., Wilson A.G., Moskowitz R., Hochberg M., Loeser R., Hooper M., Renner J.B., Crane M.M. (2007). The Genetics of Generalized Osteoarthritis (GOGO) study: Study design and evaluation of osteoarthritis phenotypes. Osteoarthr. Cartil..

[50] Kellgren J.H., Lawrence J.S. (1957). Radiological assessment of osteo-arthrosis. Ann. Rheum. Dis..

[51] American Rheumatism Association Glossary Committee (1982). Dictionary of the rheumatic diseases. Signs Symptoms.

[52] Devlin B., Roeder K., Wasserman L. (2001). Genomic control, a new approach to genetic-based association studies. Theor. Popul. Biol..

[53] Yin L., Zhang H., Tang Z., Xu J., Yin D., Zhang Z., Yuan X., Zhu M., Zhao S., Li X. (2020). rMVP: A Memory-efficient, Visualization-enhanced, and Parallel-accelerated tool for Genome-Wide Association Study. BioRxiv.

[54] Solé X., Guinó E., Valls J., Iniesta R., Moreno V. (2006). SNPStats: A web tool for the analysis of association studies. Bioinformatics.

[55] Barrett J.C., Fry B., Maller J., Daly M.J. (2005). Haploview: Analysis and visualization of LD and haplotype maps. Bioinformatics.

[56] Watanabe K., Taskesen E., van Bochoven A., Posthuma D. (2017). Functional mapping and annotation of genetic associations with FUMA. Nat. Commun..

[57] Rentzsch P., Witten D., Cooper G.M., Shendure J., Kircher M. (2019). CADD: Predicting the deleteriousness of variants throughout the human genome. Nucleic Acids Res..

[58] Boyle A.P., Hong E.L., Hariharan M., Cheng Y., Schaub M.A., Kasowski M., Karczewski K.J., Park J., Hitz B.C., Weng S. (2012). Annotation of functional variation in personal genomes using RegulomeDB. Genome Res..

[59] Nomura A., Tada H., Teramoto R., Konno T., Hodatsu A., Won H.-H., Kathiresan S., Ino H., Fujino N., Yamagishi M. (2016). Whole exome sequencing combined with integrated variant annotation prediction identifies a causative myosin essential light chain variant in hypertrophic cardiomyopathy. J. Cardiol..

[60] Wickham H. (2016). Ggplot2—Elegant Graphics for Data Analysis.

