# Supplementary material for: A Functional Polymorphism Downstream of Vitamin A Regulator Gene CYP26B1 Is Associated with Hand Osteoarthritis
Source: Int J Mol Sci. 2023 Feb 3;24(3):3021. doi: 10.3390/ijms24033021 (PMC9918232; doi:10.3390/ijms24033021)
Supplement: Supplementary file 1 [file ijms-24-03021-s001.zip › Supplementary Figure S1.pdf]

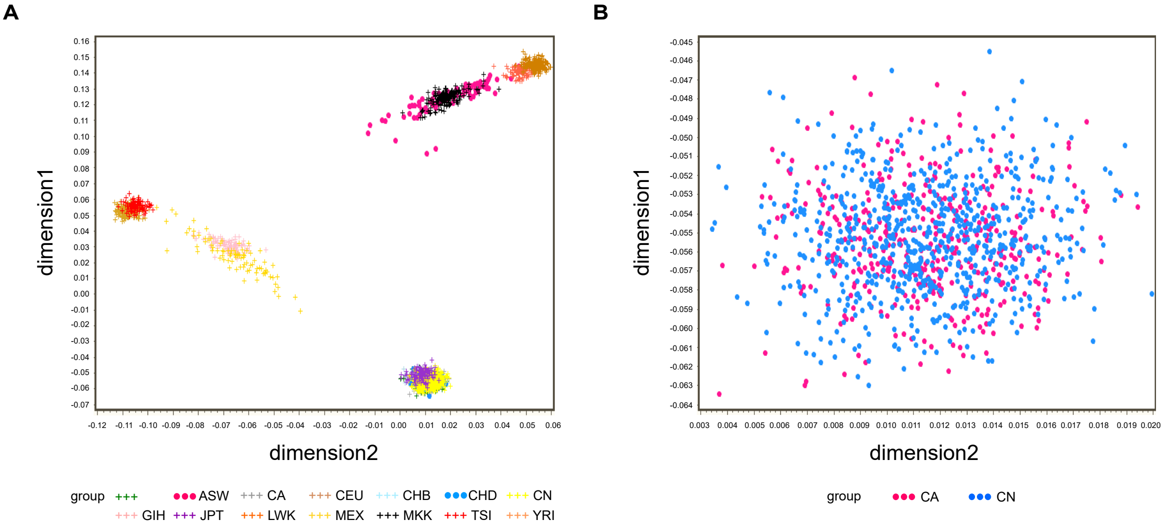


Supplementary Figure S1. Multidimensional scaling plot for the trend test. **A** The muiltidimensional scaling plot (MDS) of the GWAS samples with the HapMap population. **B** MDS of GWAS case-control. ASW, Americans with African ancestry living in Southwest USA; CEU, Utah residents with Northern and Western European ancestry from the Centre d'Etude du Polymorphisme Humain collection; CHB, Han Chinese population in Beijing, China; CHD, Chinese population in metropolitan Denver, DO; GIH, Gujarati Indian population in Houston, TX; JPT, Japanese population in Tokyo; LWK, Luhya population in Webuye, Kenya; MEX, Mexicans in Los Angeles, CA; MKK, Maasai in Kinayawa, Kenya; TSI, Tuscan population in Italy; YRI, Yoruban population in Ibadan, Nigeria; CA, hand OA cases; CN, OA-unaffected controls.
