# Supplementary material for: A Functional Polymorphism Downstream of Vitamin A Regulator Gene CYP26B1 Is Associated with Hand Osteoarthritis
Source: Int J Mol Sci. 2023 Feb 3;24(3):3021. doi: 10.3390/ijms24033021 (PMC9918232; doi:10.3390/ijms24033021)
Supplement: Supplementary file 1 [file ijms-24-03021-s001.zip › Supplementary Figure S2.pdf]

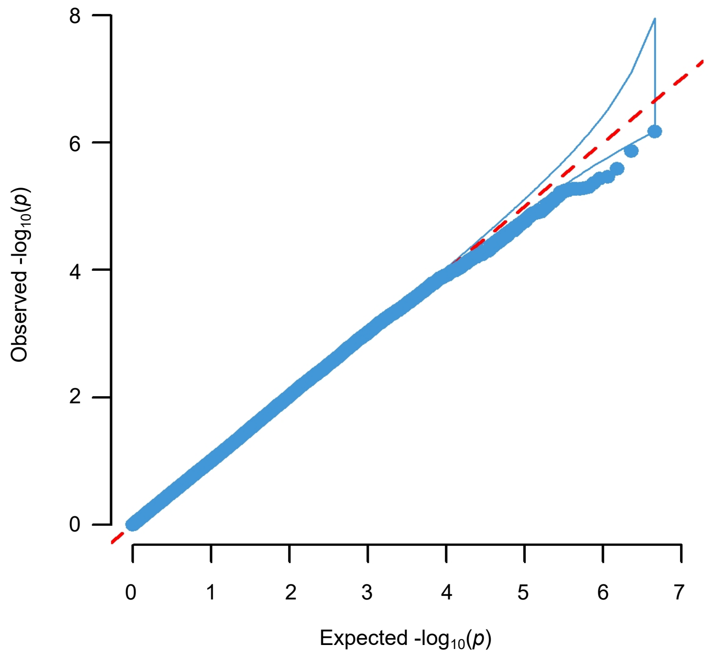

Supplementary Figure S2. Quantile-quantile plot of the observed *P*-values versus the expected null *P*-values distribution. The Q-Q plot was constructed after stringent QC analysis and imputation of 4,587,241 SNPs.
