# Supplementary material for: A Functional Polymorphism Downstream of Vitamin A Regulator Gene CYP26B1 Is Associated with Hand Osteoarthritis
Source: Int J Mol Sci. 2023 Feb 3;24(3):3021. doi: 10.3390/ijms24033021 (PMC9918232; doi:10.3390/ijms24033021)
Supplement: Supplementary file 1 [file ijms-24-03021-s001.zip › Supplementary Figure S3.pdf]

A

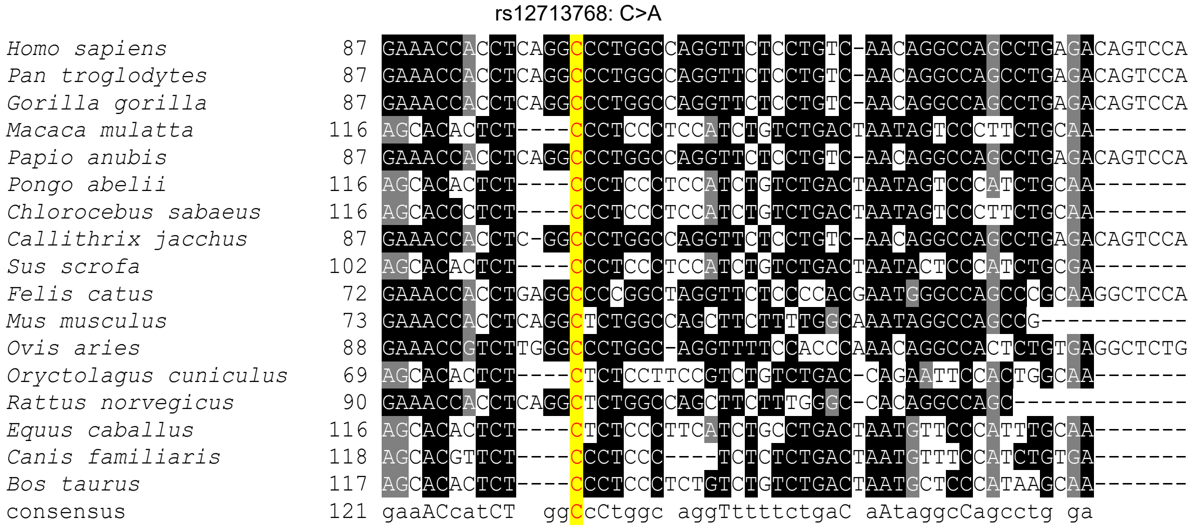

B

Organism	Chr	Identity[a]	Distance (bp)[b]	Gene[c]	Location[d]	Accession
Pan troglodytes	2A	100%	118532	LOC738157	Chr2A: 72,283,693-72,302,973	NC_036880.1
Gorilla gorilla	2A	99.50%	118598	LOC101126229	Chr2A: 88,488,277-88,506,890	NC_044603.1
Macaca mulatta	13	98.51%	104361	LOC704750	Chr13: 36,656,295-36,675,586	NC_041766.1
Papio anubis	14	98.51%	104537	*CYP26B1*	Chr14: 72,460,531-72,479,135	NC_044989.1
Pongo abelii	2A	98.01%	105239	LOC100446166	Chr2A: 36,432,062-36,452,148	NC_036904.1
Chlorocebus sabaeus	14	96.52%	97379	*CYP26B1*	Chr14: 35,030,011-35,057,164	NC_023655.1
Callithrix jacchus	14	94.55%	106747	*CYP26B1*	Chr14: 37,259,670-37,279,488	NC_048396.1
Sus scrofa	3	82.21%	104699	LOC100521659	Chr3: 70,573,551-70,593,416	NC_010445.4
Felis catus	A3	80.85%	81186	*CYP26B1*	ChrA3: 91,168,443-91,189,206	NC_018725.3
Mus musculus	6	80.83%	82692	*CYP26B1*	Chr6: 84,548,396-84,570,890	NC_000072.7
Ovis aries	3	78.43%	128627	LOC101110922	Chr3: 100,383,775-100,400,597	NC_040254.1
Oryctolagus cuniculus	2	78.06%	89698	LOC100341926	Chr2: 114,279,277-114,297,359	NC_013670.1
Rattus norvegicus	4	77.61%	94475	*CYP26B1*	Chr4: 116,261,796-116,278,615	NC_005103.4
Equus caballus	15	76.96%	97426	*CYP26B1*	Chr15: 31,695,380-31,715,089	NC_009158.3
Canis familiaris	17	74.76%	78225	*CYP26B1*	Chr17: 50,633,397-50,653,882	NC_006599.3
Bos taurus	11	74.61%	116041	*CYP26B1*	Chr11: 12,371,161-12,396,520	NC_037338.1

Chr, chromosome.
[a]similarity retrieved from 100bp flanking sequences up- and downstream of SNP rs12713768 compared to human
[b]distance from C allele of rs12713768 to the coding sequence of *CYP26B1* gene
[c]name of *CYP26B1* gene in each organism retrieved from NCBI
[d]position of *CYP26B1* gene in each organism

Supplementary Figure S3. The risk-conferring C allele of SNP rs12713768 is **A** conserved and **B** located within 150kb from gene *CYP26B1* across 16 mammalian genomes.
